# Case Report: Two Cases of Soft-Tissue Sarcomas: High TMB as a Potential Predictive Biomarker for Anlotinib Combined With Toripalimab Therapy

**DOI:** 10.3389/fimmu.2022.832593

**Published:** 2022-05-06

**Authors:** Yong Li, Yihong Liu, Yanchun Qu, Xian Chen, Xin Qu, Yongsong Ye, Xiaohua Du, Ying Cheng, Mian Xu, Haibo Zhang

**Affiliations:** ^1^ Department of Oncology, Guangdong Provincial Hospital of Chinese Medicine, Guangzhou, China; ^2^ Department of Pathology, Guangdong Provincial Hospital of Chinese Medicine, Guangzhou, China; ^3^ Department of Medical, Shanghai OrigiMed Co., Ltd, Shanghai, China

**Keywords:** soft-tissue sarcomas, tumor mutational burden (TMB), immune checkpoint inhibitors, anlotinib, toripalimab

## Abstract

Soft-tissue sarcomas (STS), with over 100 different histologic subtypes, are rare tumors that account for 1% of all adult malignancies. Immune checkpoint inhibitors (ICIs) display certain benefits in some subtypes, especially in undifferentiated pleomorphic sarcoma (UPS), alveolar soft part sarcoma (ASPS), and leiomyosarcoma (LMS). However, efficacy is difficult to predict. High tumor mutational burden (TMB-H) and programmed death-ligand 1 (PD-L1) expression are the strongest features associated with the efficacy of immunotherapy, although they are rarely found in STS patients. Until now, whether or not PD-L1 expression and TMB are related to the efficacy of immunotherapy has not been determined. In this study, we report data obtained from two STS patients, one ASPS and one UPS with a high TMB, that benefited from anlotinib combined with toripalimab following resistance to anlotinib monotherapy. A 26 year-old female patient was diagnosed with ASPS. PD-L1 was negative. Next generation sequencing (NSG) revealed *ASPSCR1-TFE3* fusion and TMB-H. Following eight months of anlotinib monotherapy, the patient’s disease progressed but continued to benefit from subsequent use of anlotinib combined with toripalimab for 19 months. Another 63 year-old male patient was diagnosed with UPS. PD-L1 was positive and NGS revealed TMB-H. Following 19 months of anlotinib monotherapy, the patient’s disease progressed but continued to benefit from subsequent use of anlotinib combined with toripalimab. DFS is 23 months to follow-up time. The results presented are the first to report the relationship between TMB and the efficacy of immunotherapy in STS. Based on our results, we hypothesis that anlotinib combined with toripalimab is effective for the treatment of some advanced ASPS or UPS. TMB may be a potential predictive biomarker for ICI treatment and deserves additional study.

## Introduction

Soft-tissue sarcomas (STS) are a rare and heterogeneous group of tumors that represent less than one percent of all adult malignancies (1). STS contains more than 100 different histologic and molecular subtypes, each of which exhibits different clinical manifestations. Treatments include chemotherapy, targeted therapy, and immunotherapy. Since some subtypes of STS are chemoresistant, chemosensitivity is dependent on specific histologic type. Novel target drugs have been approved, including pazopanib, regorafenib, and anlotinib (approved in China) (2–4). Immune checkpoint inhibitors (ICIs) have indicated initial efficacy in some subtypes, especially in myxofibrosarcoma, undifferentiated pleomorphic sarcoma (UPS), alveolar soft part sarcoma (ASPS), cutaneous angiosarcoma, undifferentiated sarcomas, and leiomyosarcoma (5–8). ICIs plus anti-VEGR therapy have indicated preliminary activity, particularly in patients with ASPS (9). However, these therapies are still not perfect and predictive biomarkers are unclear. Tumor mutational burden (TMB) and programmed death-ligand 1 (PD-L1) expression are the strongest features associated with the efficacy of immunotherapy treatment, but are rarely found in STS patients. As such, identifying patients that will benefit from ICI treatment is crucial.

In this study, we report data obtained from one ASPS and one UPS patient with high TMB (TMB-H) following anlotinib resistance that received anlotinib combined with toripalimab therapy, and achieved a partial response (PR) and stable disease (SD), respectively. Our data suggest that TMB-H may be a potential predictive biomarker for immunotherapy.

## Case 1

**Figure 1:**
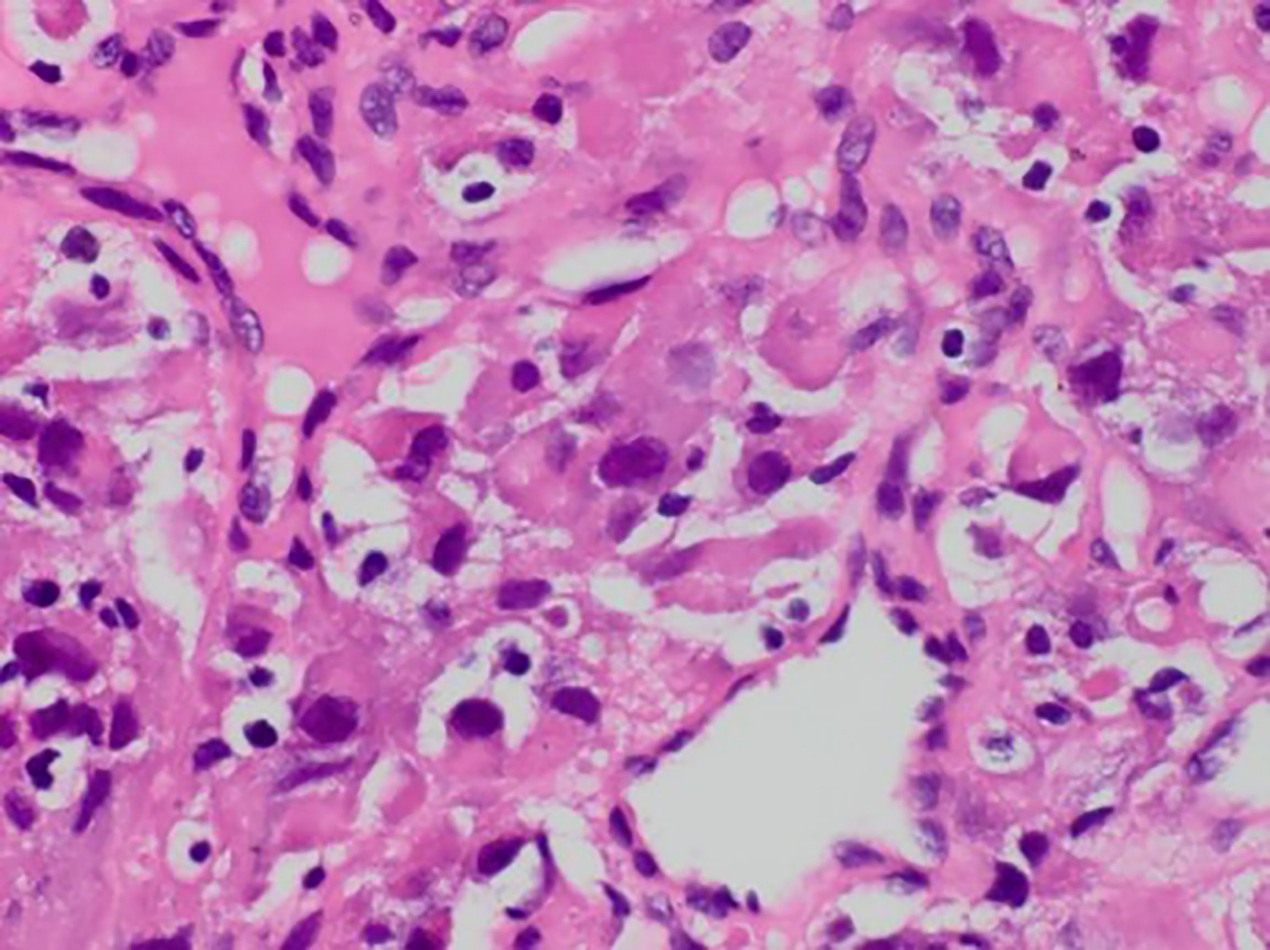
Postoperative pathology revealed obvious heterogeneity in tumor cells with hyperchromatic, irregular nuclei and a foamy cytoplasm (HE, 400x).

**Figure 2:**
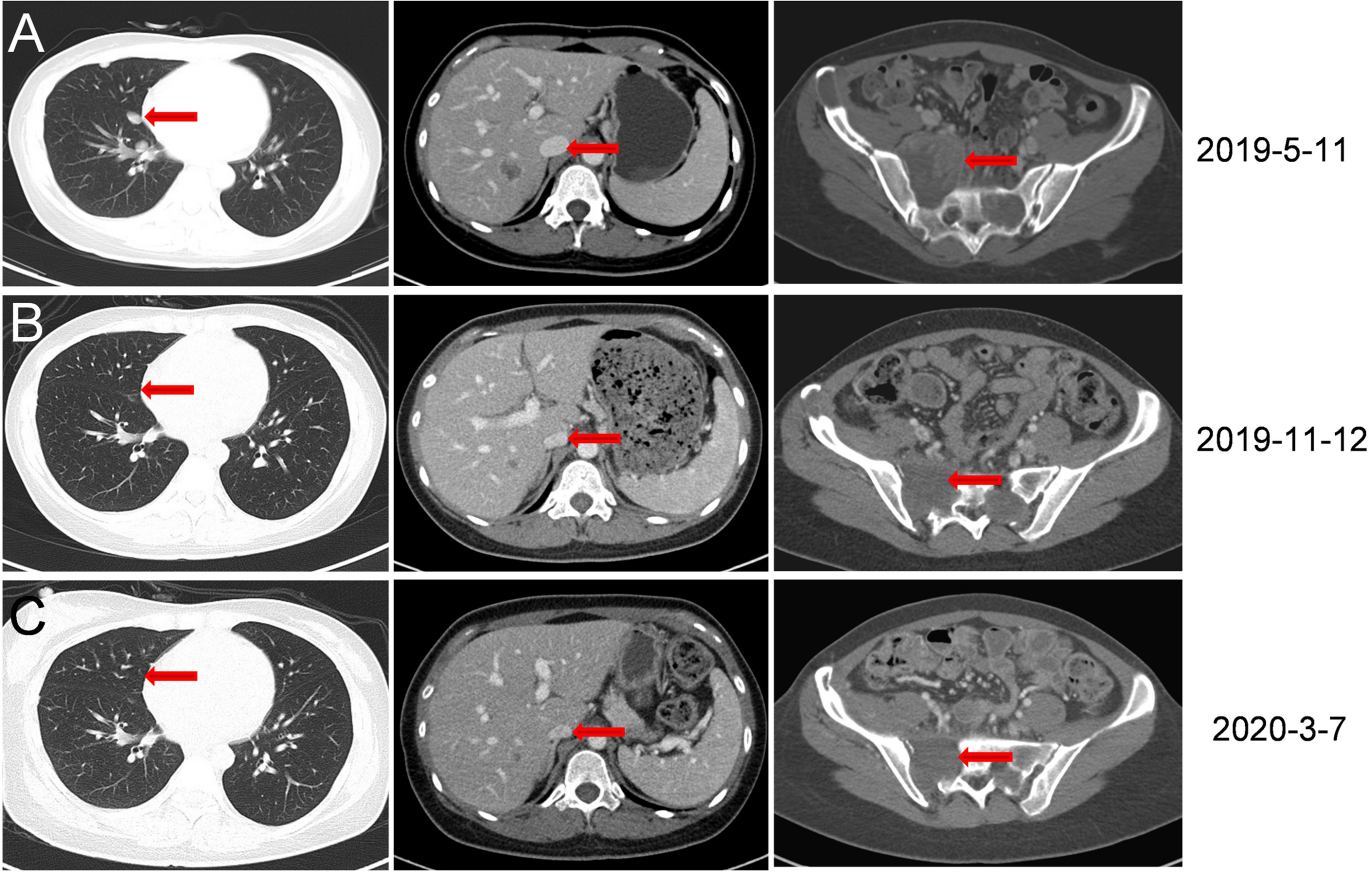
CT scan images during treatment. **(A)** In May 2019, eight months following anlotinib treatment, new multiple lung metastases were observed. **(B)** In November 2019, six months following anlotinib combined with toripalimab treatment, lesions in the lung disappeared. Liver and sacral vertebrae metastases shrank. (Size was reduced from 5.5 cm × 5.4 cm to 3.5 cm × 3.8 cm). **(C)** In March 2020, CT re-examination indicated stable disease.

**Figure 3:**
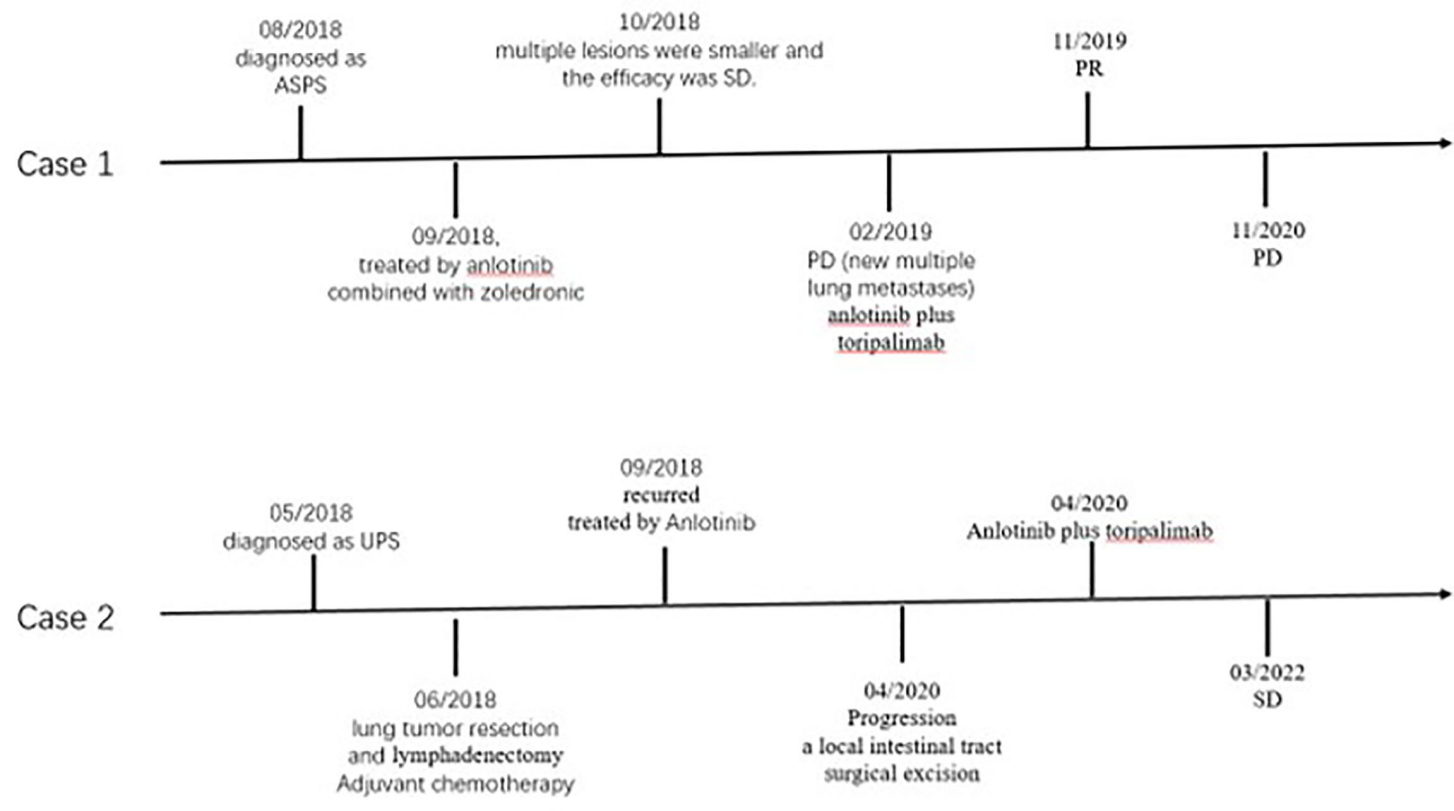
Schematic picture showing the time line of treatment procedure.

In July 2018, a 26 year-old female patient underwent bone emission computed tomography (ECT), positron emission tomography (PET), and magnetic resonance imaging (MRI) examinations for bone pain in our hospital. These examinations revealed a right thigh mass and abnormal activation of multiple focal bone metabolism, indicating a malignant bone tumor with metastases. In August 2018, a biopsy of tumor mass and a sacroiliac joint biopsy were performed. The pathological diagnosis report indicated that the mass on the outer right thigh was consistent with alveolar soft part sarcoma (ASPS) (**Figure 1**). Additionally, next generation sequencing (NGS) of biopsy tissue yielded 13.1 muts/Mb of TMB, determined as TMB-Hbased on the previous data (10) andpositive ASPSCR1-TFE3 fusion. Immunohistochemistry (IHC) assay using 28-8 antibody showed tumor cells were PD-L1 negative. The neutrophil-to-lymphocyte ratio (NLR) was 2.17. In September 2018, anlotinib (12 mg, qd, d1-14, q3w) combined with zoledronic acid was used to treat the tumor. A PET/CT re-examination in October 2018 showed that multiple lesions were smaller than previously recorded. According to RECIST 1.1, the efficacy was stable disease (SD). However, in February 2019, following eight months of treatment, disease progression (PD) was observed (new multiple lung metastases occurred, **Figure 2A**), and soreness of the shoulder and upper arm was reported to be aggravated. Based on previous treatment and NGS results, a treatment of anlotinib combined with toripalimab (240 mg, ivd, d1, q3w, a PD-1 blocker drug) began in May 2019. Soreness was relieved after one week. In November 2019, CT re-examination indicated that the efficacy evaluation was a partial response (PR) (**Figure 2B**). Anlotinib combined with toripalimab was continued as treatment, and re-examination in March 2020 suggested that the disease was stable (**Figure 2C**). Bone metastasis was SD, and ostalgia worsened in July 2020. However, pain was reduced after replacing bisphosphonates with denosumab. A CT scan revealed that the disease was PD in December 2020. Progression free survival (PFS) was 19 months (**Figure 3**).

## Case 2

**Figure 4:**
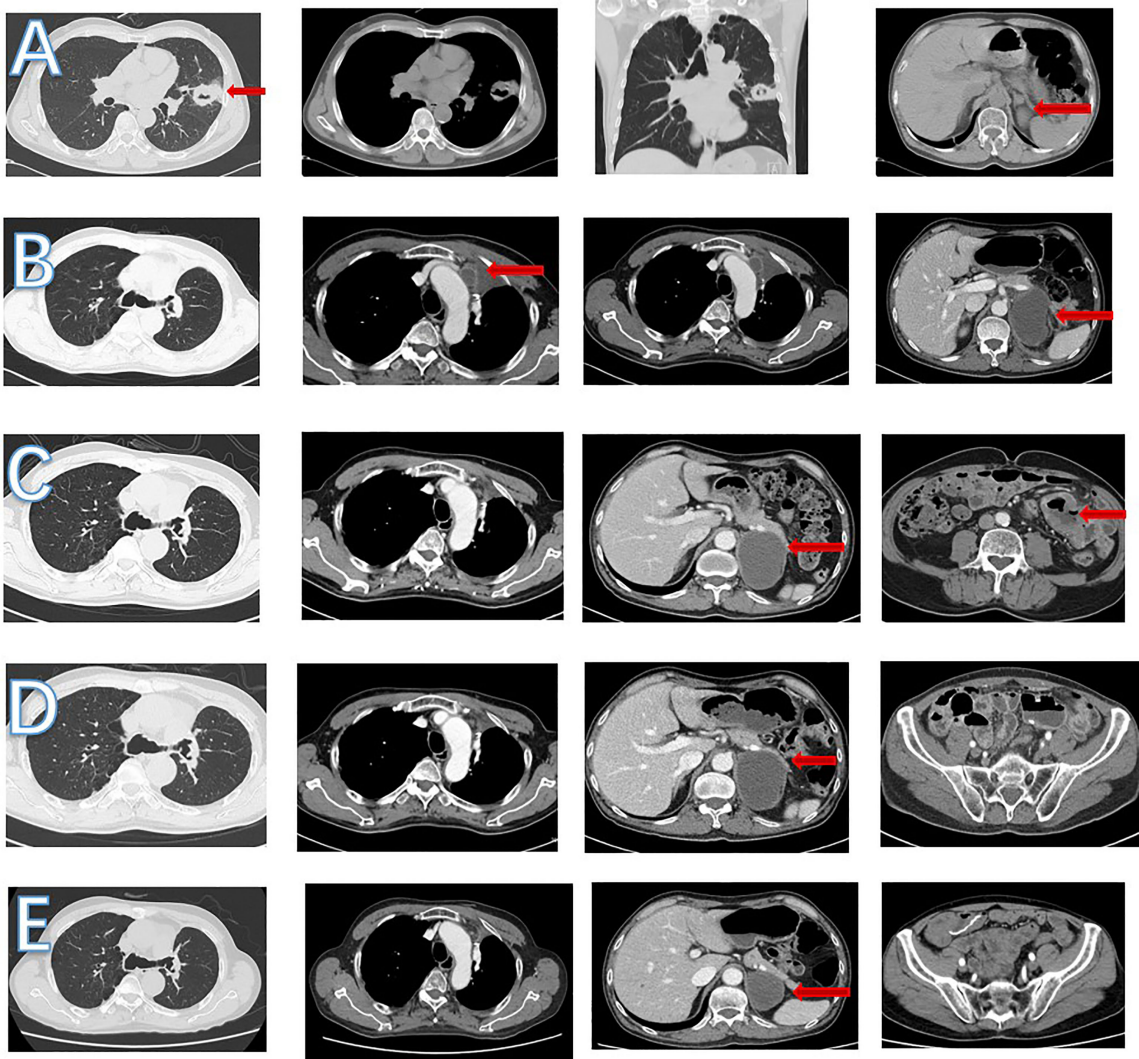
CT scan images during treatment. **(A)** In June 2018, left lung nodules and suspected metastasis of left adrenal nodule. **(B)** In September 2018, enlarged mediastinal lymph nodule and increased left adrenal nodule. **(C)** In April 2020, a new small bowel mass was found, left adrenal nodule was stable and mediastinal lymph nodule shrank. **(D)** In July 2020, left adrenal and mediastinal lymph nodule was stable. No small intestinal tumor was found. **(E)** In March 2022, left adrenal nodule shrank and mediastinal lymph nodule was stable.

**Figure 5:**
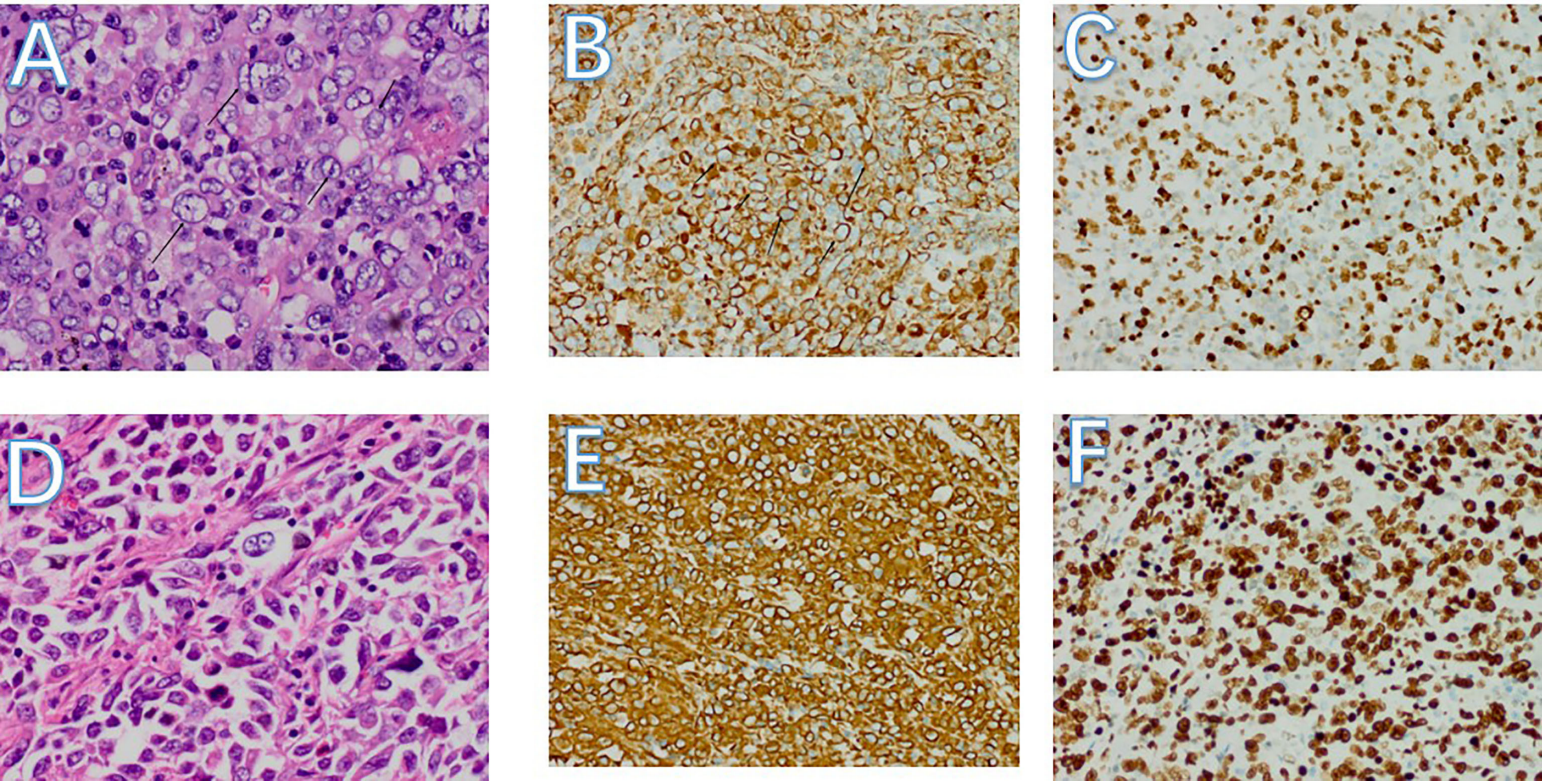
**(A-C)** Pathology of left lung mass. **(A)** Pulmonary tumors with cytologic atypia,different nuclear sizes. Most of the nuclei are vacuolated, some nucleoli can be seen, and mitotic figures are obvious (HE, 400x). **(B)** Diffuse cytoplasmic positivity in tumor cells (Vimentin 200x). C:tumor cells are nuclear-positive, showing a hyperproliferative state. (ki67 200x) **(D-F)** Pathology of small bowel mass. **(D)** Small bowel tumors with different nuclear sizes. Most of the nuclei are vacuolated, some nucleoli can be seen, and mitotic figures are obvious (HE, 400x). **(E)** Diffuse cytoplasmic positivity in tumor cells (Vimentin 200x). **(F)** Tumor cells are nuclear-positive, showing a hyperproliferative state. (ki67 200x).

In May 2018, a 63 year-old male patient came to our hospital due to cough and blood-streaked sputum. A CT scan revealed left lung nodules and lymphadenectasis in the mediastinum and left hilar and suspected metastasis of left adrenal nodule (**Figure 4A**). The clinical stage was T2bN2Mx. The patient underwent a left upper lung tumor resection combined with a lymphadenectomy in June 2018. Intraoperative rapid cryopathology was performed and the pathological diagnosis was lung adenocarcinoma. However, postoperative pathology confirmed the pathological diagnosis was UPS (**Figure 5**). Immunohistochemistry showed vimentin was positive, and TTF1, CK5/6, and CK7 were negative. Surgical tissues were sent for NGS testing. The results of NGS indicated TMB-H (10.0 muts/Mb) (10). PD-L1 expression in tumor (using the 28-8 IHC assay) was 90%. The patient received pemetrexed plus platinum as adjuvant chemotherapy. The tumor recurred three months later (**Figure 4B**). Anlotinib was then administered orally at once, and the efficacy was SD. However, following 19 months of the treatment, the patient presented with abdominal pain and a small bowel mass was found, indicating tumor progression (**Figure 4C**). In April 2020, the other lesions are stable, therefore a local intestinal tract surgical excision was performed and the pathology showed UPS (**Figure 5**). Anlotinib combined with toripalimab treatment (240 mg, ivd, d1, q3w) was then administered. In July 2020, a CT re-examination indicated that tumors had shrunk (**Figure 4D**), and the efficacy evaluation was SD. Treatment with combination therapy is currently ongoing. In March 2022, a CT re-examination indicated the efficacy was SD (**Figure 4E**) and PFS is 23 months (until the time of manuscript submission) (**Figure 3**).

## Discussion

ICIs, consisting of PD-L1, programmed cell death protein-1 (PD-1), and cytotoxic T-lymphocyte associated protein 4 (CTLA-4) inhibitors, have revolutionized the treatment of human solid tissue tumors (11). Some studies indicate that ICIs are also effective in STS (5–7), although the results are heterogeneous for different subtypes. Monotherapy pembrolizumab in a Phase 2 clinical trial indicated that seven (18%) of 40 patients had an objective response for four (40%) of ten patients with UPS, two (20%) of ten patients with liposarcoma, and one (10%) of ten patients with synovial sarcoma. In another clinical trial of 38 patients treated with nivolumab and ipilimumab, the confirmed objective response rate (ORR) was 16% and demonstrated promising efficacy for certain sarcoma subtypes (UPS, LMS, myxofibrosarcoma, and angiosarcoma) (6). Higher levels of efficacy were determined for gene expression related to antigen presentation and T-cell infiltration in UPS and LMS, as compared to synovial sarcoma and liposarcoma (12). These outcomes partly explain the positive results for UPS and LMS. However, the exact reason why ICIs is effective remained elusive.

Targeted therapy for sarcoma is largely achieved with small-molecule, multi-target, anti-vascular drugs, including pazopanib, regorafenib and anlotinib, approved for the treatment of different types of sarcomas (2–4). Single-agent tyrosine kinase inhibitor (TKI) drugs are effective for the treatment of some sarcomas. Combined treatments maybe improve efficacy. In recent years, TKIs combined with ICIs have attracted extensive attention. In a single-arm clinical trial, the median PFS of the ASPS subgroup treated with axitinib plus pembrolizumab was 12.4 months, and the evaluated 3-month progression-free survival for ASPS was 72.7% (9), and the ORR was 60.0%, indicating that TKIs combined with ICIs may have preliminary activity in patients with advanced sarcomas, particularly in patients with ASPS (9).

ASPS is an extremely rare subtype of STS, with only one diagnosis per 10 million people per year, accounting for approximately 0.5% of all sarcomas (13). Although ASPS is often reported as an indolent disease as compared to other subtypes of sarcoma, many patients have distant metastases or multiple local recurrences at diagnosis; and the prognosis is poor (14). Conventional chemotherapy has little therapeutic influence on ASPS (15). Molecular analyses of ASPS has revealed a specific ASPSCR1-TFE3 gene fusion that causes the upregulation of angiogenesis and proliferation transcripts (16), providing evidence for the application of TKIs directed at vascular endothelial growth factor (VEGF). Anlotinib, pazopanib, and sunitinib have been recommended as a first-line therapy for ASPS (4, 17, 18). Additionally, ICIs have shown good efficacy for ASPS (19). For example, the PD-L1 inhibitor atezolizumab has been shown to achieve a 42% objective response (20). Toripalimab is a recombinant humanized anti-PD-1 monoclonal antibody, independently developed in China. In a Phase I clinical trial (JS001) of toripalimab for advanced or refractory ASPS, one and two of 12 total patients achieved CR and PR, respectively. The median PFS (mPFS) is expected to be 12.4 months (21). For this trial, the ORR, disease control rate (DCR), and median overall survival (OS) were 25.0% (3/12), 91.7% (11/12), and 34.7 months, respectively, in ASPS patients with toripalimab treatment (22). The combination of TKIs and ICIs have been investigated in advanced sarcomas (9). One possibility in explaining the therapeutic effect of the immune checkpoint blockade in ASPS is ASPSCR1-TFE3 fusion. Such fusion could theoretically serve as a highly selective tumor-specific antigen (23).

Immunotherapy has shown preliminary influence in the treatment of sarcoma, however, clear predictive biomarkers remain uncertain. PD-L1 expression andTMB-H can predict the efficacy of immunotherapy in most tumors (24, 25), although no affirmation has been drawn for STS. Some studies suggest that specific subtypes of UPS and LMS sarcoma may predict the response to immunotherapy (7). However, PD-L1 status, TMB, and microsatellite instability status have not been investigated.

A past study investigated the expression of PD-L1 in sarcoma and found a positive rate of only 12%; one positive outcome resulted from a radiation associated pleomorphic sarcoma and the other from a spindle cell sarcoma (26). Another study indicated intra-tumoral infiltration of PD1-positive lymphocytes and PD-L1 expression (no tumor expression) in 65% and 58% of STS, respectively (27); patients with a PD1 (+)/PD-L1 (+) pattern had the shortest survival period. In the SARC028 clinical trial, PD-L1 was at the 1% threshold in only three (4%) of 70 patients and all three patients had UPS. Of the three positive patients, only two had a response: one had a complete response and the other had a partial response (5). In a trial of axitinib plus pembrolizumab, tumor cell PD-L1 expression was positive in 15 (52%) of 29 patients, although neither PD-L1 positivity nor an increased tumor-infiltrating lymphocyte score correlated with progression-free survival of longer than six months, or achieved a partial response. A baseline neutrophil-to-lymphocyte ratio of more than five is associated with a progressive response (9). Therefore, the expression of PD-L1 cannot predict the efficacy of immunotherapy in some STS patients. However, due to the lack of data and the heterogeneity of sarcoma, such a conclusion is contradictory. Our second patient with high PD-L1 expression combined with TMB-H suggests that PD-L1 expression may be a positive biomarker for predicting efficacy in UPS patients.

TMB is often detected in various tumors and is related to the efficacy of ICI treatment (28). Yet, the TMB of STS has been reported to be low (**Table 1**) (34, 35). A study indicated that the TMB of 68 STS patients was relatively low (median: 2.05 per Mb) (36). Previous studies have also indicated that ASPS displays minimal differences in mutations (9). However, both of our patients with TMB-H benefit from anlotinib combined with toripalimab treatment, suggesting that TMB may be a potential biomarker for therapy. A previously published case report found that a UPS patient with high TMB was effectively treated with a single anti-PD-1 agent (37). Some cases show the same result (**Table 2**). As such, our results indicate that combination therapy may be effective in anti-vascular, therapy-resistant patients with TMB-H.

**Table 1:** TMB status in STS.

Author	Number	Pathological pattern	TMB	Reference
He M, etc.	16	synovial sarcoma	1/16 (6.25%)TMB-High(this case is dMMR)	(29)
Cancer Genome Atlas Research Network	205	adult soft tissue sarcomas	Average TMB is 1.06perMb, 2/205 is TMB-H(Two cases are dMMR),	(30)
Xu L, etc.	199	soft-tissue or osteogenic sarcomas	median TMB was 1.5 muts/Mb (range, 0.7–24.5 muts/Mb). 5/199 is TMB-H,1 with fibrosarcoma, 2 with angiosarcoma and 2 patients with unclassified soft-tissue sarcoma	(31)
Kim H, etc.	60	sarcoma	1.7% (1/60) TMBH,no dMMR	(32)
Casey DL, etc.	87	Rhabdomyosarcoma	median TMB was 1.1 (range, 0–13.8), 22/87 was TMB-H (defined as the top quartile or≥2.8 muts/Mb)	(33)
Chalmers ZR, etc.	157	soft tissue angiosarcoma,	median TMB was 3.8 muts/Mb, 13.4% (8.9–19.6)of cases had more than 20 mutations/Mb	(34)
Chalmers ZR, etc.	260	Soft tissue sarcoma undifferentiated	median TMB was 2.5 muts/Mb, 8.1% (5.3-12) of cases had more than 20 mutations/Mb	(34)

dMMR, mismatch‐repair deficient; muts/Mb, mutations/Mb.

**Table 2:** Efficacy and TMB status in STS.

Author	Report type	Number	Pathological pattern	Regimen	TMB	Efficacy	Reference
Zheng S, etc.	Case Report	1	osteosarcoma	chemotherapy and subsequent PD-1 inhibitor	High	continuous remission for 33 months	(38)
Cheung LS, etc.	Case Report	1	undifferentiated pleomorphic sarcomas	anti-PD-1 therapy	High	complete response, DFS:22 months to follow-up time	(37)
Cheung LS, etc.	Case Report	1	undifferentiated pleomorphic sarcomas	anti-PD-1 and anti-CTLA-4	High	stable brain and bone lesions for the last 18 months	(37)
Luo Y, etc.	Case Report	1	high-grade myxofibrosarcoma	PD-1 inhibitor	positive PD-L1 and TMB was 215Muts	partial response. DFS 18 months to follow-up time	(39)
Vieira AC, etc.	Case Report	1	metastatic cutaneous sarcoma	PD-1 inhibitor	29 Muts/Mb	partial response. DFS 28 cycles	(40)

DFS, disease free survival.

An Open-Label Phase IB trial found that the combination of toripalimab plus axitinib is tolerable and displays promising antitumor activity in patients with treatment-naive metastatic mucosal melanoma (41). The finding suggests that toripalimab combined with anti-vascular targeted drugs may have a synergistic effect. Another case report also indicated that the combination of ICIs with anti-VEGF has satisfactory curative effect (42, 43). Similarly, for our cases, following resistance to anlotinib monotherapy and by considering TMB-H, our STS and the ASPS patient were both treated with anlotinib combined with toripalimab and reached SD and PR, respectively.

To our knowledge, this is the first report for the combination of anti-VEGF and PD-L1 inhibition for TMB-H STS. Our results are encouraging. However, the following limitations exist for our results : 1. We could not confirm whether or not the effect was due to a single immunotherapy or to the synergy of combined treatment. 2. Our case reports only provide preliminary conclusions, although they, at least, indicated that TMB-H may be a potential biomarker. 3. Specific reasons for effectiveness of the combined treatment remains elusive.

Large randomized controlled trials should be conducted in order to examine the potential benefits of the PD-1/PD-L1 blockade, either alone or in combination with targeted therapy. To determine patients most likely to benefit from ICIs; and to evaluate TMB, the tumor microenvironment, gene expression, and genetic alterations, as well as to explore underlying cellular and molecular mechanisms, future studies are warranted.

## Conclusion

Anlotinib combined with toripalimab was shown to be an effective therapy in advanced STS or ASPS patients with metastases, specifically for TMB-H STS. Our study indicates that anti-VEGF TKIs combined with ICIs may be an effective choice for the treatment of STS or ASPS. The two cases examined suggest that TMB-H may be a potential predictor. This hypothesis will need to be tested in a larger clinical trial that encompasses robust correlative studies. The ongoing clinical trial NCT02609984, NCT02815995, and NCT02636725 will confirm the efficacy of ICIs and explore predictive marker.

## Data Availability Statement

The raw data supporting the conclusions of this article will be made available by the authors, without undue reservation.

## Ethics Statement

The studies involving human participants were reviewed and approved by Ethics committee of Guangdong Provincial Hospital of Chinese Medicine. The patients/participants provided their written informed consent to participate in this study.

## Author Contributions

Conception/Design: YL and HZ; Provision of study material or patients: YL and YHL; Collection and/or assembly of data: YQ, XC, and XQ; Data analysis and interpretation: YY, XD, and YC; Manuscript writing: YL and MX; Final approval of manuscript: YL and HZ. All authors have read and approved the submitted version of the manuscript.

## Conflict of Interest

Authors MX and YC are employed by Shanghai OrigiMed Co., Ltd.

The remaining authors declare that the research was conducted in the absence of any commercial or financial relationships that could be construed as a potential conflict of interest.

## Publisher’s Note

All claims expressed in this article are solely those of the authors and do not necessarily represent those of their affiliated organizations, or those of the publisher, the editors and the reviewers. Any product that may be evaluated in this article, or claim that may be made by its manufacturer, is not guaranteed or endorsed by the publisher.
